# A potential relationship between *MMP-9* rs2250889 and ischemic stroke susceptibility

**DOI:** 10.3389/fneur.2023.1178642

**Published:** 2023-07-05

**Authors:** Hanming Ge, Xiaojuan Ma, Jiachen Wang, Xiaobo Zhang, Yu Zhang, Qi Zhang, Wu Li, Jie Liu, Jinwei Duan, Wenzhen Shi, Ye Tian

**Affiliations:** ^1^Department of Neurology, Xi'an Key Laboratory of Cardiovascular and Cerebrovascular Diseases, Xi'an No. 3 Hospital, The Affiliated Hospital of Northwest University, Xi'an, Shaanxi, China; ^2^Medical Research Center, Xi'an Key Laboratory of Cardiovascular and Cerebrovascular Diseases, Xi'an No. 3 Hospital, The Affiliated Hospital of Northwest University, Xi'an, Shaanxi, China; ^3^The College of Life Sciences, Northwest University, Xi'an, Shaanxi, China

**Keywords:** MMP-9, SNP, susceptibility, case-control study, ischemic stroke

## Abstract

**Purpose:**

Ischemic stroke (IS), a serious cerebrovascular disease, greatly affects people's health and life. Genetic factors are indispensable for the occurrence of IS. As a biomarker for IS, the *MMP-9* gene is widely involved in the pathophysiological process of IS. This study attempts to find out the relationship between *MMP-9* polymorphisms and IS susceptibility.

**Methods:**

A total of 700 IS patients and 700 healthy controls were recruited. The single nucleotide polymorphism (SNP) markers of the *MMP-9* gene were genotyped by the MassARRAY analyzer. Multifactor dimensionality reduction (MDR) was applied to generate SNP–SNP interaction. Furthermore, the relationship between genetic variations (allele and genotype) of the *MMP-9* gene and IS susceptibility was analyzed by calculating odds ratios (ORs) and 95% confidence intervals (CIs).

**Results:**

Our results demonstrated that rs2250889 could significantly increase the susceptibility to IS in the codominant, dominant, overdominant, and log-additive models (*p* < 0.05). Further stratification analysis showed that compared with the control group, rs2250889 was associated with IS risk in different case groups (age, female, smoking, and non-drinking) (*p* < 0.05). Based on MDR analysis, rs2250889 was the best model for predicting IS risk (cross-validation consistency: 10/10, OR = 1.56 (1.26–1.94), *p* < 0.001).

**Conclusion:**

Our study preliminarily confirmed that SNP rs2250889 was significantly associated with susceptibility to IS.

## 1. Introduction

Stroke, an acute cerebrovascular disease, is the main cause of death and long-term disability (1). The National Epidemiological Survey of Stroke in China (NESS-China) estimated that the death rate of stroke in China was 149.49 per 100,000, accounting for 1.57 million deaths in 2018 (2). There are two main types of strokes: ischemic stroke (IS) and hemorrhagic stroke (HS). Previous research studies have suggested that IS has become a major disease in China and one of the most important reasons for people's disabilities (3). From this perspective, IS remains one of the biggest challenges that China is facing, and the need for a large number of research studies on the pathogenesis of IS is more than justified.

Although the pathogenesis of IS still has been debated, more and more studies have highlighted that the pathogenesis of IS is regulated by multiple factors, such as smoking, hypertension, diabetes, drinking, and age (4). Recently, a report of Mendelian randomization has identified supported atrial fibrillation (AF), body mass index (BMI), smoking, blood pressure, white matter hyperintensity, and type 2 diabetes mellitus (T2DM) as risk factors of IS (5). In addition, many studies have emphasized the influence of genetic factors on IS, and exactly, single nucleotide polymorphisms (SNPs) in gene have a certain regulatory effect on the occurrence of IS (4, 6). For example, *ALDH7A1* rs12514417 polymorphism may increase the risk of IS in individuals exposed to alcohol (7). *OPG* SNP T245G is associated with enhanced IS risk among the Chinese (8). At present, the research on post-genome-wide association study (GWAS) is ongoing, which mainly studies whether genetic variants associated with different disease phenotypes exert their pathogenicity, and it is a means of exploring the relationship between gene polymorphisms and disease risk.

Matrix metalloproteinases (MMPs) are a group of zinc-dependent endoproteinases that regulate extracellular matrix (ECM) *via* proteolysis, cell adhesion, and cytokines (9). In addition, MMPs have been proven to participate in a series of biological processes, such as cell proliferation, migration, angiogenesis, and immune response (10, 11). MMP family members include MMP-1, -2, -3, -7, -8, -9, -12, -13, MT1-MMP, and MT3-MMP, which are expressed in various vascular tissues and cells (12). The matrix metalloproteinase-9 (*MMP-9*) gene is located in 20q13.12 and consists of 13 exons and 12 introns. It plays a role in the movement of vascular smooth muscle cells and the instability of atherosclerotic plaque (13, 14). At present, a meta-analysis showed that *MMP-9* and *MMP-12* gene polymorphisms may be risk factors for IS, whereas *MMP-1, MMP-2*, and *MMP-3* were not associated with the risk of IS (15). Moreover, the latest study has found that rs243849 and rs14070 in *MMP-2* are significantly related to the risk of IS in the Shaanxi population from China (16). However, no significant association between *MMP-9* genetic variants and intracerebral hemorrhage (ICH) susceptibility was reported (17). We hypothesized that *MMP-9* gene variants may play different roles in the occurrence of IS. Therefore, our case–control study aimed to investigate the relationship between *MMP-9* SNPs and the susceptibility to IS in the Northwest Chinese population to predict potential loci for IS risk.

## 2. Methods

### 2.1. Research participants

The study participants, including 700 IS patients and 700 healthy controls, were consecutively recruited from Xi'an No. 3 Hospital from August 20 to December 22. The case group consisted of IS patients, who were admitted to the hospital within 72 h after the symptom appeared and had focal neurological deficit symptoms lasting for more than 24 h. According to the diagnostic criteria of the World Health Organization (WHO), all IS patients were confirmed by at least two independent neurologists using brain computed tomography (CT) scans, magnetic resonance imaging (MRI), and blood routine tests. Participants in the control group were normal and healthy, without other complicated cerebrovascular diseases, tumors, hypertension, diabetes, and other medical histories. All study participants were from the Northwest of China and provided written informed consent.

### 2.2. Primer design

By retrieving the NCBI (http://www.ncbi.nlm.nih.gov/omim/) and GeneBank databases combined with the MassArray analysis software, the primer sequence was designed. These primers were sent to Shangon Biotechnology Co., Ltd (Shanghai, China) to synthesize for the polymerase chain reaction (PCR) process of *MMP-9* SNPs (Supplementary Table 1).

### 2.3. Gene selecting and SNP genotyping

The physical position of *MMP-9* on chromosome 20:46008908–46016561 was obtained through the e!GRCh37 (http://asia.ensembl.org/Homo_sapiens/Info/Index) database, and the table of MMP-9 variants was downloaded. SNPs were selected based on Hardy–Weinberg equilibrium (HWE) > 0.01, MAF > 0.05, and Min Genotype > 75% using Haploview software. We further combined MassARRAY primer design software, HWE > 0.05, MAF > 0.05, and the call rate > 95% in our study population, for the selection of SNPs. Among the remaining SNPs, three candidate SNPs (rs2250889, rs17577, and rs13925) in MMP-9 have previously been reported to be associated with stroke risk (18, 19) but have not been studied in a northwest Chinese Han population. Hence, this study was conducted to explore the correlation between these SNPs and IS risk in the northwest Chinese Han population. The dbSNP, Haploreg, and RegulomeDB databases were used for predicting the potential function of these SNPs.

Approximately 5 ml of peripheral blood samples were collected from each participant and placed in tubes containing ethylenediaminetetraacetic acid (EDTA) in a −80°C refrigerator. Subsequently, genomic DNA was extracted from blood samples using the GoldMag nanoparticles method (Gold Mag Co. Ltd., Xi'an, China) according to the manufacturer's instructions. NanoDrop 2000 (Thermo Scientific, Waltham, Massachusetts, USA) was used to determine the concentration and purity of DNA. Genotyping was performed in real-time using the MassARRAY software (v. 3.0.0.4). Data management and analysis were performed by the Agena MassARRAY analysis design software (v.4).

### 2.4. Statistical analysis

All statistical analyses in our study were performed using SPSS (v.22). The differences in demographic characteristics between IS patients and controls were estimated by the chi-square test for categorical variables and the student's *t*-test for continuous variables. HWE was assessed using the χ^2^ test with one degree of freedom. The strength of all associations between genetic variants in *MMP-9* and IS risk was evaluated by logistic regression models with odds ratios (ORs) with 95% confidence intervals (CIs). Statistical analysis was performed using the online SNPStats (v.9.4) and PLINK (v.1.09) analysis software. Logistic regression analysis was introduced to study the effect of each characteristic on the relationship between *MMP-9* variants and IS risk. The credibility of the significant associations was assessed by false-positive report probability (FPRP) analysis, with 0.2 as an FPRP threshold and 0.1 as a prior probability (20, 21). Multivariate dimension reduction (MDR) software was utilized to further evaluate SNP-SNP interaction and predict the best model related to IS risk (22). All *p*-values are two-sided, with a *p*-value of < 0.05 indicating statistical significance, whereas the corrected *p*-value of < 0.05/3 was considered statistically significant after the Bonferroni correction.

## 3. Results

### 3.1. Study information about the case–control group

The detailed demographic characteristics of the case and control groups that were included are shown in Table 1. A total of 1,400 subjects were selected, including 700 IS patients and 700 healthy controls. There were no significant differences between the two groups in gender, age, smoking status, and drinking status (*p* > 0.05).

**Table 1:** General characteristics of IS patients and healthy subjects.

**Variables**	**Cases (*n* = 700)**	**Controls (*n* = 700)**	***p***
**Age, years (mean** ±**SD)**^a^			**0.095**
≤ 55	302 (43.1%)	391 (49.5%)	
>55	398(56.9%)	309(44.1%)	
**Gender** ^b^			**0.911**
Male	459(65.6%)	457 (65.3%)	
Female	241 (34.4%)	243 (34.7%)	
**Smoking status** ^a^			**0.957**
Smoking	337 (48.1%)	339(48.3%)	
Non-smoking	363(51.9%)	361(51.7%)	
**Drinking status** ^a^			**0.708**
Drinking	337 (48.1%)	345 (49.3%)	
Non-drinking	363(51.9%)	355 (50.7%)	
**Hypertension complications**			
Yes	461 (65.9%)		
No	239 (34.1%)		
**Diabetes complications**			
Yes	169 (24.1%)		
No	531 (75.9%)		

IS, ischemic stroke. *p*^a^, Student's t-test is used. *p*^b^, *P*earson's χ^2^ test is used. *p* < 0.05 indicates statistical significance.

### 3.2. Results of *MMP-9* gene polymorphism and IS risk

**Figure 1:**
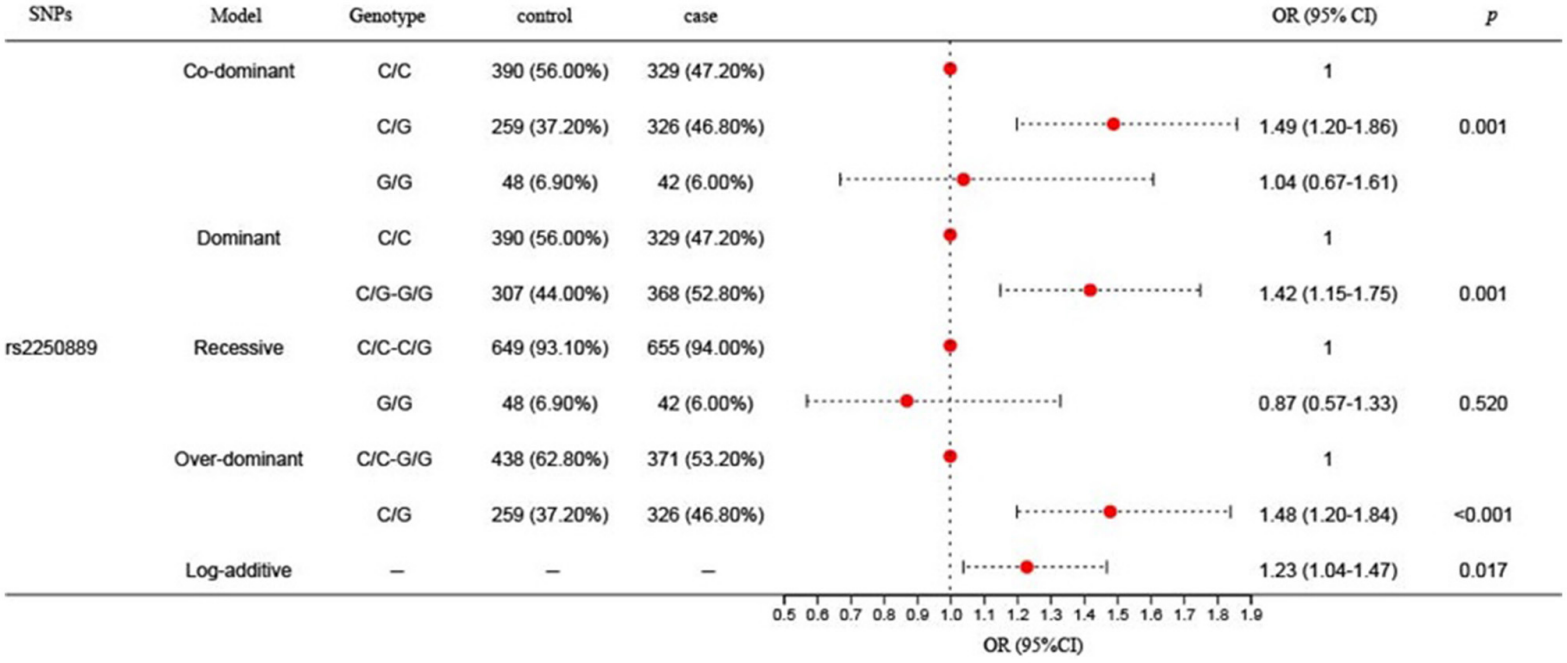
Association results of *MMP-9* gene polymorphism rs2250889 with stroke risk. SNPs, single nucleotide polymorphisms; CI, confidence interval; MAF, minor allele frequency; OR, odds ratio. *p*^a^: Student's *t*-test is used; *p* < 0.05 indicates statistical significance.

Three SNPs (rs2250889, rs17577, and rs13925) of *MMP-9* were successfully screened, and the basic information about them was demonstrated (Table 2). Database analysis presented that the potential functions of these SNPs might be related to promoter/histone marks, DNAse, protein-bound motifs changed, eQTL, and transcription factor binding. In addition, the association between three SNPs in *MMP-9* and IS susceptibility was analyzed under five genetic models (codominant, dominant, recessive, overdominant, and log-additive), as illustrated in Figure 1. We found that rs2250889 significantly increased susceptibility to IS under the codominant (CG vs. CC, OR = 1.49, 95% CI = 1.20–1.86, *p* = 0.001), dominant (CG-GG vs. CC, OR = 1.42, 95% CI = 1.15–1.75, *p* = 0.001), overdominant (CC-CG vs. GG, OR = 1.48, 95% CI = 1.20–1.84, *p* = 0.0003), and log-additive (OR = 1.49, 95% CI = 1.20–1.86, *p* = 0.017) models. However, no significant association of rs17577 and rs13925 with IS risk was revealed according to our statistical results. The results are presented in Supplementary Table 2. The significant association between rs2250889 and IS risk under the codominant, dominant, and overdominant models still existed after Bonferroni correction.

**Table 2:** Basic information and allele frequencies among *MMP-9* SNPs.

**SNP ID**	**Alleles (minor/major)**	**Chromosome position**	**MAF**		**O (HET)**	**E (HET)**	***p*^a^-for HWE**	**OR (95% CI)**	***p* ^b^**	**dbSNP func annot**	**HaploReg v4.1**	**RegulomeDB**
			**Case**	**Control**								
rs2250889	G/C	20:46013767	0.294	0.254	0.372	0.380	0.618	1.22 (1.03–1.44)	**0.020**	Missense R (Arg) > P (Pro)	Promoter histone marks, Enhancer histone marks, DNAse, Proteins bound, Motifs changed	eQTL/caQTL + TF binding + matched TF motif + matched Footprint + chromatin accessibility peak
rs17577	A/G	20:46014472	0.125	0.124	0.223	0.218	0.727	1.01 (0.81–1.27)	0.909	Missense R (Arg) > Q (Gln)	Promoter histone marks, Enhancer histone marks, DNAse, Proteins bound, Selected eQTL hits	eQTL/caQTL + TF binding + any motif + Footprint + chromatin accessibility peak
rs13925	A/G	20:46016326	0.124	0.127	0.222	0.221	1.000	0.98 (0.79–1.23)	0.875	Synonymous V (Val) > V (Val)	Enhancer histone marks, Motifs changed, Selected eQTL hits	eQTL/caQTL + TF binding/chromatin accessibility peak

Chr, chromosome; CI, confidence interval; HWE, Hardy-Weinberg equilibrium; MAF, minor allele frequency; OR, odds ratio; SNPs, single nucleotide polymorphisms. dbSNP (https://www.ncbi.nlm.nih.gov/snp/); Haploreg (https://pubs.broadinstitute.org/mammals/haploreg/haploreg.php); RegulomeDB (https://regulome.stanford.edu/regulome-search/). *p*^a^: Student's t-test is used. *p*^b^: *P*earson's χ^2^ test is used. Bold *p* < 0.05 indicates statistical significance.

### 3.3. Stratification analysis by demographic characteristics

The stratified analysis by gender (male and female), age, drinking, and smoking was conducted to further analyze the correlation between selected SNPs and IS risk, as shown in Table 3. The effect of rs2250889 on the risk of IS varied obviously with age. More precisely, rs2250889 played a risk-increasing role in IS in individuals aged ≤ 55 years under four genetic models (codominant: OR = 1.64, *p* = 0.009; dominant: OR = 1.57, *p* = 0.004; overdominant: OR = 1.61, *p* = 0.002; and log-additive: OR = 1.33, *p* = 0.026) and among people older than 55 years old, the risk-increasing effect of rs2250889 on IS susceptibility under two models (dominant: OR = 1.42, *p* = 0.045 and overdominant: OR = 1.53, *p* = 0.017) was observed. Furthermore, rs2250889 was associated with an increased susceptibility to IS in women (codominant: OR = 2.13, *p* = 0.001; dominant: OR = 1.91, *p* = 0.001; overdominant: OR = 2.11, *p* = 0.000; log-additive: OR = 1.44, *p* = 0.017) but not statistically different in men. Ultimately, the analysis stratified by smoking status revealed that rs2250889 exerted a risk-increasing effect on IS in smokers (codominant: OR = 1.58, *p* = 0.011; dominant: OR = 1.48, *p* = 0.014; and overdominant: OR = 1.61, *p* = 0.003). Among the non-drinking subjects, SNP rs2250889 was significantly correlated with IS susceptibility under four models (codominant: OR = 2.04, *p* = 0.0001; dominant: OR = 1.93, *p* **<** 0.0001; overdominant: OR = 1.95, *p* **<** 0.0001; and log-additive: OR = 1.54, *p* = 0.0005). After Bonferroni correction, rs2250889 was associated with an increased IS risk in the subgroup aged ≤ 51 years (codominant, dominant, and overdominant), women (codominant, dominant, and overdominant), smokers (codominant, dominant, and overdominant), and non-drinkers (codominant, dominant, overdominant, and log-additive), respectively. Unfortunately, there were no significant associations between other SNPs (rs17577, rs13925) and IS risk in terms of age, gender, smoking, and drinking (Supplementary Table 3).

**Table 3:** Significant results of *MMP-9* rs2250889 on IS susceptibility under the stratification of demographic characteristics (age, gender, smoking and drinking status).

**Model**	**Genotype**	**Control**	**Case**	**OR (95% CI)**	***P***	**Control**	**Case**	**OR (95% CI)**	***P***
**Age**		>**55 years**				≤ **55 years**			
Codominant	C/C	173 (56.50%)	195 (49.20%)	1		217 (55.50%)	134 (44.50%)	1	
	C/G	111 (36.30%)	177 (44.70%)	1.52 (1.06–2.17)	0.058	148 (37.90%)	149 (49.50%)	1.64 (1.19–2.25)	**0.009** ^*^
	G/G	22 (7.20%)	24 (6.10%)	0.94 (0.46–1.92)		26 (6.70%)	18 (6.00%)	1.14 (0.60–2.19)	
Dominant	C/C	173 (56.50%)	195 (49.20%)	1		217 (55.50%)	134 (44.50%)	1	
	C/G-G/G	133 (43.50%)	201 (50.80%)	1.42 (1.01–2.01)	**0.045**	174 (44.50%)	167 (55.50%)	1.57 (1.15–2.13)	**0.004** ^*^
Recessive	C/C-C/G	284 (92.80%)	372 (93.90%)	1		365 (93.30%)	283 (94.00%)	1	
	G/G	22 (7.20%)	24 (6.10%)	0.78 (0.39–1.57)	0.488	26 (6.70%)	18 (6.00%)	0.91 (0.48–1.71)	0.770
Overdominant	C/C-G/G	195 (63.70%)	219 (55.30%)	1		243 (62.10%)	152 (50.50%)	1	
	C/G	111 (36.30%)	177 (44.70%)	1.53 (1.08–2.17)	**0.017**	148 (37.90%)	149 (49.50%)	1.61 (1.18–2.20)	**0.002** ^*^
Log-additive	-	-	-	1.22 (0.92–1.62)	0.169	-	-	1.33 (1.03–1.71)	**0.026**
**Sex**		**Female**				**Male**			
Codominant	C/C	143 (58.90%)	105 (43.90%)	1		247 (54.40%)	224 (48.90%)	1	
	C/G	79 (32.50%)	118 (49.40%)	2.13 (1.43–3.17)	**0.001** ^*^	180 (39.60%)	208 (45.40%)	1.27 (0.97–1.67)	0.220
	G/G	21 (8.60%)	16 (6.70%)	1.08 (0.52–2.25)		27 (6.00%)	26 (5.70%)	1.01 (0.57–1.81)	
Dominant	C/C	143 (58.90%)	105 (43.90%)	1		247 (54.40%)	224 (48.90%)	1	
	C/G-G/G	100 (41.10%)	134 (56.10%)	1.91 (1.31–2.79)	**0.001** ^*^	207 (45.60%)	234 (51.10%)	1.24 (0.95–1.61)	0.120
Recessive	C/C-C/G	222 (91.40%)	223 (93.30%)	1		427 (94.00%)	432 (94.30%)	1	
	G/G	21 (8.60%)	16 (6.70%)	0.78 (0.39–1.59)	0.490	27 (6.00%)	26 (5.70%)	0.91 (0.51–1.60)	0.730
Overdominant	C/C-G/G	164 (67.50%)	121 (50.60%)	1		274 (60.40%)	250 (54.60%)	1	
	C/G	79 (32.50%)	118 (49.40%)	2.11 (1.44–3.10)	**< 0.001** ^*^	180 (39.60%)	208 (45.40%)	1.27 (0.97–1.66)	0.081
Log-additive	-	-	-	1.44 (1.07–1.93)	**0.017**	-	-	1.14 (0.91–1.42)	0.240
**Smoking**		**Smokers**				**Non-smokers**			
Codominant	C/C	197 (58.30%)	162 (48.50%)	1		193 (53.80%)	167 (46.00%)	1	
	C/G	122 (36.10%)	158 (47.30%)	1.58 (1.15–2.19)	**0.011** ^*^	137 (38.20%)	168 (46.30%)	1.36 (0.99–1.86)	0.160
	G/G	19 (5.60%)	14 (4.20%)	0.83 (0.39–1.75)		29 (8.10%)	28 (7.70%)	1.15 (0.65–2.03)	
Dominant	C/C	197 (58.30%)	162 (48.50%)	1		193 (53.80%)	167 (46.00%)	1	
	C/G-G/G	141 (41.70%)	172 (51.50%)	1.48 (1.08–2.03)	**0.014** ^*^	166 (46.20%)	196 (54.00%)	1.32 (0.98–1.79)	0.066
Recessive	C/C-C/G	319 (94.40%)	320 (95.80%)	1		330 (91.90%)	335 (92.30%)	1	
**Age**		>**55 years**				≤ **55 years**			
	G/G	19 (5.60%)	14 (4.20%)	0.68 (0.33–1.41)	0.300	29 (8.10%)	28 (7.70%)	1.00 (0.57–1.74)	1.000
Overdominant	C/C-G/G	216 (63.90%)	176 (52.70%)	1		222 (61.80%)	195 (53.70%)	1	
	C/G	122 (36.10%)	158 (47.30%)	1.61 (1.17–2.21)	**0.003** ^*^	137 (38.20%)	168 (46.30%)	1.33 (0.99–1.81)	0.062
Log-additive	-	-	-	1.26 (0.97–1.64)	0.088	-	-	1.19 (0.94–1.51)	0.150
**Drinking**		**Drinkers**				**Non-drinkers**			
Codominant	C/C	179 (51.90%)	168 (50.00%)	1		211 (59.90%)	161 (44.60%)	1	
	C/G	144 (41.70%)	151 (44.90%)	1.06 (0.77–1.45)	0.590	115 (32.70%)	175 (48.50%)	2.04 (1.48–2.81)	**0.0001** ^*^
	G/G	22 (6.40%)	17 (5.10%)	0.74 (0.37–1.47)		26 (7.40%)	25 (6.90%)	1.44 (0.79–2.63)	
Dominant	C/C	179 (51.90%)	168 (50.00%)	1		211 (59.90%)	161 (44.60%)	1	
	C/G-G/G	166 (48.10%)	168 (50.00%)	1.01 (0.74–1.38)	0.930	141 (40.10%)	200 (55.40%)	1.93 (1.42–2.62)	**< 0.001** ^*^
Recessive	C/C-C/G	323 (93.60%)	319 (94.90%)	1		326 (92.60%)	336 (93.10%)	1	
	G/G	22 (6.40%)	17 (5.10%)	0.72 (0.37–1.41)	0.330	26 (7.40%)	25 (6.90%)	1.06 (0.59–1.89)	0.850
Overdominant	C/C-G/G	201 (58.30%)	185 (55.10%)	1		237 (67.30%)	186 (51.50%)	1	
	C/G	144 (41.70%)	151 (44.90%)	1.09 (0.80–1.49)	0.590	115 (32.70%)	175 (48.50%)	1.95 (1.42–2.66)	**< 0.001** ^*^
Log-additive	-	-	-	0.96 (0.74–1.24)	0.760	-	-	1.54 (1.20–1.96)	**0.0005** ^*^

IS, ischemic stroke; OR, odds ratio; CI, confidence interval. Bold *p* < 0.05 indicates statistical significance. ^*^p indicate that after Bonferroni correction (*p* < 0.05/3) means the data is statistically significant.

### 3.4. Stratification analysis by complications

The relationship between *MMP-9* SNPs and IS risk was also investigated in the subgroups of IS patients with/without hypertension or diabetes, as shown in Table 4 and Supplementary Table 4. Compared with healthy controls, rs2250889 was related to the risk of IS in IS patients with hypertension (codominant: OR = 1.54, *p* = 0.002; dominant: OR = 1.47, *p* = 0.002; overdominant: OR = 1.53, *p* = 0.001; and log-additive: OR = 1.26, *p* = 0.018) and IS patients without hypertension (overdominant: OR = 1.42, *p* = 0.022). Moreover, rs2250889 was found to be related to an increased risk of IS in IS patients without diabetes (codominant: OR = 1.55, *p* = 0.001; dominant: OR = 1.46, *p* = 0.001; overdominant: OR = 1.55, *p* < 0.001; and log-additive: OR = 1.24, *p* = 0.024). After Bonferroni correction, rs2250889 might be a risk factor for IS patients with hypertension (codominant, dominant, and overdominant) and IS patients without diabetes (codominant, dominant, and overdominant). However, other SNPs (rs17577, rs13925) were not significantly associated with IS risk in terms of hypertension and diabetes.

**Table 4:** Significant results of *MMP-9* rs2250889 on IS susceptibility under the stratification of complications (hypertension and diabetes).

**Model**	**Genotype**	**Control**	**Case1**	**Case2**	**OR (95% CI)**	***P***	**OR (95% CI)**	***P***	**OR (95% CI)**	***P***
			**IS with HYP**	**IS without HYP**	**IS with HYP vs. Control**		**IS without HYP vs. Control**		**IS with HYP vs. Stroke without HYP**	
Codominant	C/C	390 (56%)	213 (46.4%)	116 (48.7%)	1	**0.002** ^*^	1	0.072	1	0.760
	C/G	259 (37.2%)	218 (47.5%)	108 (45.4%)	1.54 (1.20–1.97)		1.42 (1.04–1.93)		1.13 (0.81–1.56)	
	G/G	48 (6.9%)	28 (6.1%)	14 (5.9%)	1.07 (0.65–1.76)		0.98 (0.52–1.85)		1.11 (0.56–2.21)	
Dominant	C/C	390 (56%)	213 (46.4%)	116 (48.7%)	1	**0.002** ^*^	1	0.047	1	0.460
	C/G-G/G	307 (44%)	246 (53.6%)	122 (51.3%)	1.47 (1.16–1.86)		1.35 (1.00–1.82)		1.13 (0.82–1.55)	
Recessive	C/C-C/G	649 (93.1%)	431 (93.9%)	224 (94.1%)	1	0.610	1	0.590	1	0.890
	G/G	48 (6.9%)	28 (6.1%)	14 (5.9%)	0.88 (0.54–1.43)		0.84 (0.46–1.56)		1.05 (0.54–2.04)	
Overdominant	C/C-G/G	438 (62.8%)	241 (52.5%)	130 (54.6%)	1	**0.001** ^*^	1	**0.022**	1	0.500
	C/G	259 (37.2%)	218 (47.5%)	108 (45.4%)	1.53 (1.20–1.94)		1.42 (1.05–1.92)		1.11 (0.81–1.53)	
Log-additive	—	—	—	—	1.26 (1.04–1.52)	**0.018**	1.18 (0.93–1.49)	0.170	1.09 (0.84–1.42)	0.500
			**IS with diabetes**	**IS without diabetes**	**IS with diabetes vs. control**		**IS without diabetes vs. control**		**IS with diabetes vs. IS without diabetes**	
Codominant	C/C	390 (56%)	82 (48.8%)	247 (46.7%)	1	0.270	1	**0.001** ^*^	1	0.480
	C/G	259 (37.2%)	73 (43.5%)	253 (47.8%)	1.34 (0.94–1.91)		1.55 (1.22–1.96)		0.88 (0.61–1.27)	
	G/G	48 (6.9%)	13 (7.7%)	29 (5.5%)	1.22 (0.63–2.36)		0.96 (0.59–1.57)		1.35 (0.66–2.76)	
Dominant	C/C	390 (56%)	82 (48.8%)	247 (46.7%)	1	0.110	1	**0.001** ^*^	1	0.690
	C/G-G/G	307 (44%)	86 (51.2%)	282 (53.3%)	1.32 (0.94–1.85)		1.46 (1.16–1.83)		0.93 (0.65–1.33)	
Recessive	C/C-C/G	649 (93.1%)	155 (92.3%)	500 (94.5%)	1	0.830	1	0.330	1	0.310
	G/G	48 (6.9%)	13 (7.7%)	29 (5.5%)	1.07 (0.57–2.04)		0.79 (0.49–1.27)		1.44 (0.72–2.87)	
Overdominant	C/C-G/G	438 (62.8%)	95 (56.5%)	276 (52.2%)	1	0.130	1	**< 0.001** ^*^	1	0.370
	C/G	259 (37.2%)	73 (43.5%)	253 (47.8%)	1.31 (0.93–1.84)		1.55 (1.23–1.96)		0.85 (0.60–1.21)	
Log-additive	—	—	—	—	1.20 (0.92–1.56)	0.180	1.24 (1.03–1.49)	**0.024**	1.01 (0.76–1.36)	0.940

IS, ischemic stroke; OR, odds ratio; CI, confidence interval; HYP, hypertension. Bold *p* < 0.05 indicates statistical significance. ^*^p indicate that after Bonferroni correction (*p* < 0.05/3) means the data is statistically significant.

### 3.5. FPRP analysis of the positive findings

FPRP analysis was performed to confirm whether the significant findings deserved attention (Table 5). At the prior probability level of 0.01, the significant association of *MMP-9* rs2250889 with IS risk remained noteworthy in the overall analysis (FPRP = 0.074, 0.125, and 0.070). Moreover, the association of *MMP-9* rs2250889 with IS risk was also positive in the subgroup aged ≤ 55 years, women, smokers, and non-drinkers at the prior probability level of 0.01.

**Table 5:** False-positive report probability for the associations of *MMP-9* rs2250889 with IS risk.

**Group/SNPs ID**	**Model**	**OR (95% CI)**	**Prior probability**				
			**0.25**	**0.1**	**0.01**	**0.001**	**0.0001**
**Overall**							
	Codominant	1.49 (1.20–1.86)	**0.002**	**0.007**	**0.074**	0.448	0.890
	Dominant	1.42 (1.15–1.75)	**0.004**	**0.013**	**0.125**	0.590	0.935
	Overdominant	1.48 (1.20–1.84)	**0.002**	**0.007**	**0.070**	0.432	0.884
	Log-additive	1.23 (1.04–1.47)	**0.065**	**0.173**	0.696	0.959	0.996
**Age** > **55 years**							
	Codominant	1.52 (1.06–2.17)	**0.119**	0.288	0.816	0.978	0.998
	Dominant	1.42 (1.01–2.01)	**0.188**	0.410	0.884	0.987	0.999
	Overdominant	1.53 (1.08–2.17)	**0.101**	0.252	0.788	0.974	0.997
**Age** ≤ **55 years**							
	Codominant	1.64 (1.19–2.25)	**0.022**	**0.063**	0.425	0.882	0.987
	Dominant	1.57 (1.15–2.13)	**0.028**	**0.025**	**0.087**	**0.002**	**0.004**
	Overdominant	1.61 (1.18–2.20)	**0.081**	**0.071**	0.222	**0.005**	**0.012**
	Log-additive	1.33 (1.03–1.71)	0.491	0.457	0.758	**0.048**	**0.120**
**Females**							
	Codominant	2.13 (1.43–3.17)	0.907	0.895	0.969	0.338	0.579
	Dominant	1.91 (1.31–2.79)	0.990	0.988	0.997	0.837	0.932
	Overdominant	2.11 (1.44–3.10)	**0.001**	**0.003**	**0.035**	0.266	0.784
	Log-additive	1.44 (1.07–1.93)	**0.068**	**0.179**	0.705	0.960	0.996
**Smokers**							
	Codominant	1.58 (1.15–2.19)	**0.046**	**0.126**	0.613	0.941	0.994
	Dominant	1.48 (1.08–2.03)	**0.078**	0.202	0.736	0.966	0.996
	Overdominant	1.61 (1.17–2.21)	**0.028**	**0.001**	**0.001**	**0.002**	**0.003**
**Non-drinkers**							
	Codominant	2.04 (1.48–2.81)	**0.080**	**0.004**	**0.004**	**0.005**	**0.010**
	Dominant	1.93 (1.42–2.62)	0.490	**0.041**	**0.044**	**0.048**	**0.097**
	Overdominant	1.95 (1.42–2.66)	0.907	0.299	0.319	0.337	0.519
	Log-additive	1.54 (1.20–1.96)	0.990	0.810	0.824	0.836	0.915

SNP, single nucleotide polymorphism; OR, odds ratio; 95% CI, 95% confidence interval. The level of false-positive report probability threshold was set at 0.2, and noteworthy findings are presented. Bold indicate that the level of false-positive report probability threshold < 0.2.

### 3.6. MDR analysis of *MMP-9* SNPs

**Figure 2:**
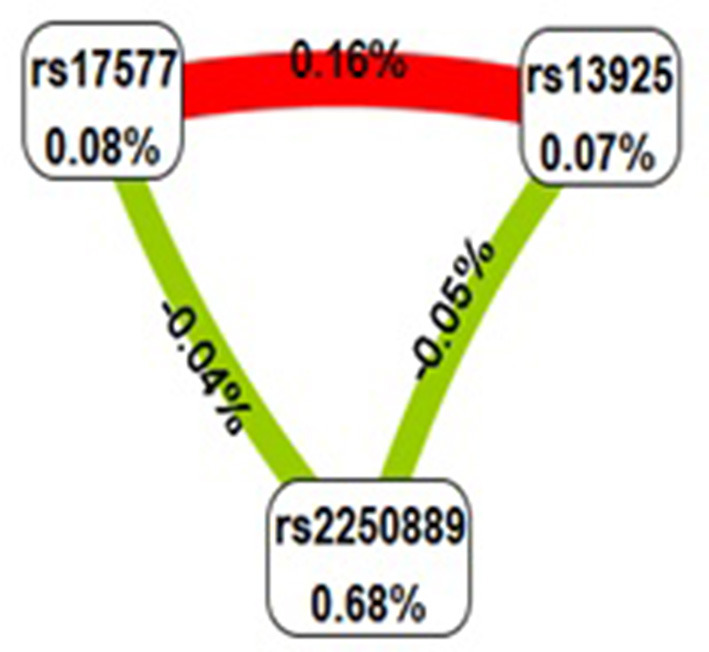
MDR analysis diagram of the *MMP-9* gene among different SNP–SNPs. A positive entropy value represents synergy, while a negative value represents redundancy. Red indicates strong synergy; fluorescent green represents a separate effect. Values (%) in nodes represent the IGs of individual attributes (main effects). Values (%) between nodes are IGs of each pair of attributes (interaction effects).

The multi-factor dimensionality reduction (MDR) analysis was carried out to find the relationship between the SNP-SNPs interactions in IS occurrence. The significant combinations of variables were selected based on the entropy measure to assess the information gain (IG) associated with attribute interaction. The patterns of entropy recapitulate the main and/or interaction effects of the paired combination of each attribute. As demonstrated in Figure 2, rs17577 and rs13925 had a synergistic effect with the IG value of 0.16%, and rs2250889 and rs17577 had strong redundancy with the IG value related to IS of −0.05%. More importantly, we found that rs2250889 was the best model for predicting IS risk in Table 6 (Cross-Validation Consistency: 10/10, OR= 1.48(1.20–1.83), *p* = 0.0003).

**Table 6:** Summary of SNP–SNP interactions on the risk of IS analyzed by MDR method.

**Model**	**Training accuracy^a^**	**Testing accuracy^a^**	**Cross-validation consistency**	**OR (95% CI)**	***p* ^a^**
rs2250889	0.548	0.546	10/10	1.48 (1.20–1.83)	**0.0003**
rs2250889, rs17577	0.552	0.522	8/10	1.50 (1.21,1.86)	**0.0002**
rs2250889, rs17577, rs13925	0.556	0.532	10/10	1.56 (1.26,1.94)	**< 0.001**

IS, ischemic stroke. Bold *p* < 0.05 indicates statistical significance; *p*^a^: *P*earson's χ^2^ test is used.

## 4. Discussion

Ischemic stroke is a type of disease that causes serious harm to the human body and cognition. It is particularly important to pay attention to clinical treatment and diagnosis. In our experiments, 1,400 subjects were recruited and statistically analyzed to report the association between *MMP-9* SNPs (rs2250889, rs17577, and rs13925) and IS susceptibility. In the overall analysis, only rs2250889 was significantly associated with IS susceptibility. In a stratified analysis, rs2250889 could increase the risk of IS in subjects of different ages, women, smokers, and non-drinkers. Moreover, rs2250889 might be a risk factor for IS patients with hypertension and IS patients without diabetes. MDR showed that rs2250889 was the best model for predicting IS risk. Taken together, our findings are the first to discuss the relationship between *MMP-9* rs2250889 and IS susceptibility and to evaluate the diagnostic significance of this SNP. Rs3749966, a missense variant [R (Arg) > P (Pro)], might be associated with promoter/histone marks, DNAse, protein-bound, motifs changed, eQTL, and transcription factor binding. Therefore, we speculated that *MMP-9* rs2250889 might influence IS occurrence by regulating the expression level of *MMP-9*, which certainly requires experimental verification.

As a member of the MMP family, *MMP-9* can regulate the balance of ECM and participate in multiple important biological processes. It has been reported that *MMP-9* is closely related to intracranial aneurysms, atherosclerosis, ischemic brain injury, and other diseases (23). Recently, studies have found that *MMP-9* polymorphism is associated with intracranial aneurysm (24). More studies have detected the expression of *MMP-9* in the serum and brain tissues of IS patients and found that it is significantly high (25, 26). In addition, the TT genotype of *MMP-9*-1562C/T has been proven to be a risk factor for hemorrhagic complications after thrombolytic therapy for acute IS (AIS) (27). More importantly, in a case–control study, *MMP-9* SNP rs3918242 was associated with the increased risk of IS in the Asian population, but there was no such finding in the Caucasian population (28). Similarly, Fan et al. have indicated that *MMP-9* rs17576 is associated with an increased IS risk in the Han Hakka population (29). Finally, our study concluded that *MMP-9* rs2250889 significantly increased the susceptibility to IS in the northwest Chinese population. This shows that the SNPs of *MMP-9* are different in different races.

Age and sex exhibit complex effects on IS risk and pathophysiology. Aging is the most important unmodifiable risk factor for IS. Compared with young stroke patients, the mortality and morbidity of elderly stroke patients are higher, and functional recovery is slower. Of note, the role of gender in IS risk is affected by age (30). Some studies have explored the impact of SNPs at different ages on IS risk in people of different ages. For instance, *PITX2* rs6817105 significantly increased the risk of stroke in people aged over 65 years (31). In this study, *MMP-9* rs2250889 was related to the increased risk of IS in subgroups of different ages. This may be due to differences in *MMP-9* SNPs. Certainly, we would not rule out a trend of getting younger of IS patients. From the perspective of gender, a recent study has revealed that young women (18–45 years old) may have a 44% higher risk of IS than their male counterparts (32). Our research also revealed that SNP rs2250889 could promote the occurrence of IS in women. Tobacco smoking can worsen IS prognosis by increasing blood–brain barrier permeability and accelerating the formation of cerebral edema (33). Drinking frequency may be also tied to the risk of IS (34). In terms of hierarchical analysis of other characteristics (smoking and drinking), similar to some studies, the genotypes rs3793917 and rs2672587 have a cumulative risk of IS in smokers (8). Our study also concluded that rs2250889 would increase the susceptibility to IS in smokers. More interestingly, the study found that *HTRA1* rs2268350 was significantly associated with IS in drinkers (8). In contrast, in this study, rs2250889 was related to the increased susceptibility to IS in non-drinkers, suggesting that rs2250889 may be a new locus on *MMP-9* for predicting IS risk.

Comorbidities are a hallmark of IS, which both increase the incidence of IS and worsen the prognosis. Hypertension is common in IS patients and the most important modifiable risk factor for IS (35). Another potential risk factor for stroke pathogenesis is diabetes mellitus, which has been shown to be associated with increased mortality in stroke (36). In this study, compared with healthy controls, rs2250889 was related to the risk of IS in IS patients with hypertension, without hypertension, and without diabetes, respectively.

Although we have discussed the results described above, several potential limitations in this study should be noted. First, our sample size is small, so we need to expand the sample size to further verify our experiment. Second, the result of this research is not generalizable as it was found in a specific group of the Chinese Han population. Third, our experiment preliminarily determined the impact of *MMP-9* on the susceptibility to IS. In subsequent experiments, we will further verify the role of *MMP-9* in the occurrence of IS through the quantitative real-time (qPCR) and Western blot (WB) experiments. Finally, IS is a complex disease. Further analysis of the impact of *MMP-9* on the pathogenesis of IS in cell and animal models is worthy of attention.

## 5. Conclusion

In summary, our research preliminarily determined the correlation between *MMP-9* gene polymorphisms (rs2250889, rs17577, and rs13925) and IS risk, especially SNP rs2250889, which provides a theoretical basis for studying the impact of *MMP-9* on the pathogenesis of IS.

## Data availability statement

The data presented in the study are deposited in the zenodo repository (https://zenodo.org/), accession number 8078657.

## Ethics statement

The studies involving human participants were reviewed and approved by Xi'an Third Hospital (Ethics Approval No.: SYXSLL-2019-034). The patients/participants provided their written informed consent to participate in this study.

## Author contributions

HG and XM completed the experiment and the original draft. JW and XZ are responsible for data processing curation. YZ, QZ, and WL took part in the collection of the sample. JL, JD, WS, and YT performed the writing—review and editing. All authors reviewed the manuscript.
