# Supplementary material for: A potential relationship between MMP-9 rs2250889 and ischemic stroke susceptibility
Source: Front Neurol. 2023 Jul 5;14:1178642. doi: 10.3389/fneur.2023.1178642 (PMC10354235; doi:10.3389/fneur.2023.1178642)
Supplement: Supplementary file 1 [file Data_Sheet_1.docx]

**Supplementary table 1** Primers used for this study.

| SNP ID | 2^nd^ - PCRP | 1^st^ - PCRP | UEP-SEQ |
| --- | --- | --- | --- |
| rs13925 | ACGTTGGATGTTCTACTGGCGCGTGAGTTC | ACGTTGGATGACTGCAGGATGTCATAGGTC | gggtAACCAGGTGGACCAAGT |
| rs2250889 | ACGTTGGATGCAAGCTGGACTCGGTCTTTG | ACGTTGGATGTTGAGCCTCCTTGACTGATG | ctgtACTCGGTCTTTGAGGAGC |
| rs17577 | ACGTTGGATGTTGGACACGCACGACGTCTT | ACGTTGGATGTAGTGTGGTGTCTCACGAAG | tttGACACGCACGACGTCTTCCAGTACC |

Abbreviations: PCRP, polymerase chain reaction primers; SNP, single‐nucleotide polymorphism; UEP-SEQ, unextended mini‐sequencing

primer. 1st-PCRP, first PCR primer; 2nd-PCRP, second PCR primer.

**Supplementary table 2** Results of the association between *MMP-9* gene polymorphism and IS susceptibility.

| SNP ID | Model | Genotype | control | case | Without adjusted | | With adjusted | |
| --- | --- | --- | --- | --- | --- | --- | --- | --- |
|  |  |  |  |  | OR (95% CI) | *P* | OR (95% CI) | *P* |
| rs17577 | Codominant | G/G | 535 (76.40%) | 529 (75.60%) | 1 |  | 1 |  |
|  |  | A/G | 156 (22.30%) | 166 (23.70%) | 1.08 (0.84-1.38) | 0.470 | 1.08 (0.84-1.39) | 0.440 |
|  |  | A/A | 9 (1.30%) | 5 (0.70%) | 0.56 (0.19-1.69) |  | 0.55 (0.18-1.65) |  |
|  | Dominant | G/G | 535 (76.40%) | 529 (75.60%) | 1 |  | 1 |  |
|  |  | A/G-A/A | 165 (23.60%) | 171 (24.40%) | 1.05 (0.82-1.34) | 0.710 | 1.05 (0.82-1.35) | 0.685 |
|  | Recessive | G/G-A/G | 691 (98.70%) | 695 (99.30%) | 1 |  | 1 | 0.269 |
|  |  | A/A | 9 (1.3%) | 5 (0.70%) | 0.55 (0.18-1.66) | 0.290 | 0.54 (0.18-1.61) |  |
|  | Overdominant | G/G-A/A | 544 (77.70%) | 534 (76.30%) | 1 |  | 1 |  |
|  |  | A/G | 156 (22.30%) | 166 (23.70%) | 1.08 (0.85-1.39) | 0.530 | 1.09 (0.85-1.40) | 0.500 |
|  | Log-additive | － | － | － | 1.01 (0.81-1.28) | 0.910 | 1.02 (0.81-1.28) | 0.893 |
| rs13925 | Codominant | G/G | 533 (76.20%) | 531 (76.10%) | 1 |  | 1 |  |
|  |  | A/G | 155 (22.20%) | 160 (22.90%) | 1.04 (0.81-1.33) | 0.610 | 1.04 (0.81-1.34) | 0.610 |
|  |  | A/A | 11 (1.60%) | 7 (1.00%) | 0.64 (0.25-1.66) |  | 0.64 (0.25-1.66) |  |
|  | Dominant | G/G | 533 (76.20%) | 531 (76.10%) | 1 |  | 1 |  |
|  |  | A/G-A/A | 166 (23.80%) | 167 (23.90%) | 1.01 (0.79-1.29) | 0.940 | 1.02 (0.79-1.30) | 0.905 |
|  | Recessive | G/G-A/G | 688 (98.40%) | 691 (99.00%) | 1 |  | 1 |  |
|  |  | A/A | 11 (1.60%) | 7 (1.00%) | 0.63 (0.24-1.64) | 0.348 | 0.63 (0.24-1.65) | 0.348 |
|  | Overdominant | G/G-A/A | 544 (77.80%) | 538 (77.10%) | 1 |  | 1 |  |
|  |  | A/G | 155 (22.20%) | 160 (22.90%) | 1.04 (0.81-1.34) | 0.740 | 1.05 (0.82-1.35) | 0.710 |
|  | Log-additive | － | － | － | 0.98 (0.78-1.23) | 0.870 | 0.99 (0.79-1.24) | 0.904 |

Abbreviations: IS, ischemic stroke; SNPs, single nucleotide polymorphisms; CI, confidence interval; MAF, minor allele frequency; OR, odds ratio.

Notes: pa: Student's t-test is used; p < 0.05 indicates statistical significance.

**Supplementary table 3** Correlations between *MMP-9* gene polymorphisms and IS susceptibility stratified by demographic characteristics (age, gender, smoking and drinking status).

| SNP ID | Model | Genotype | control | case | OR (95% CI) | *P* | control | case | OR (95% CI) | *P* |
| --- | --- | --- | --- | --- | --- | --- | --- | --- | --- | --- |
| **Age** |  |  | **> 55 years** | | | | **≤ 55 years** | | | |
| rs17577 | Codominant | G/G | 233 (75.40%) | 302 (75.90%) | 1 |  | 302 (77.20%) | 227 (75.20%) | 1 |  |
|  |  | A/G | 75 (24.30%) | 93 (23.40%) | 0.94 (0.63-1.40) | 0.850 | 81 (20.70%) | 73 (24.20%) | 1.23 (0.85-1.77) | 0.190 |
|  |  | A/A | 1 (0.30%) | 3 (0.80%) | 1.74 (0.16-19.15) |  | 8 (2.00%) | 2 (0.70%) | 0.36 (0.07-1.75) |  |
|  | Dominant | G/G | 233 (75.40%) | 302 (75.90%) | 1 |  | 302 (77.20%) | 227 (75.20%) | 1 |  |
|  |  | A/G-A/A | 76 (24.60%) | 96 (24.10%) | 0.95 (0.64-1.42) | 0.803 | 89 (22.80%) | 75 (24.80%) | 1.15 (0.80-1.65) | 0.440 |
|  | Recessive | G/G-A/G | 308 (99.70%) | 395 (99.20%) | 1 |  | 383 (98.00%) | 300 (99.30%) | 1 |  |
|  |  | A/A | 1 (0.30%) | 3 (0.80%) | 1.77 (0.16-19.41) | 0.641 | 8 (2.00%) | 2 (0.70%) | 0.34 (0.07-1.66) | 0.150 |
|  | Overdominant | G/G-A/A | 234 (75.70%) | 305 (76.60%) | 1 |  | 310 (79.30%) | 229 (75.80%) | 1 |  |
|  |  | A/G | 75 (24.30%) | 93 (23.40%) | 0.93 (0.62-1.40) | 0.730 | 81 (20.70%) | 73 (24.20%) | 1.25 (0.86-1.80) | 0.240 |
|  | Log-additive | － | － | － | 0.97 (0.66-1.42) | 0.875 | － | － | 1.06 (0.76-1.48) | 0.720 |
| rs13925 | Codominant | G/G | 232 (75.10%) | 302 (75.90%) | 1 |  | 301 (77.20%) | 229 (76.30%) | 1 |  |
|  |  | A/G | 74 (23.90%) | 92 (23.1%) | 0.95 (0.64-1.43) | 0.750 | 81 (20.80%) | 68 (22.70%) | 1.12 (0.77-1.62) | 0.510 |
|  |  | A/A | 3 (1.00%) | 4 (1.00%) | 1.97 (0.29-13.25) |  | 8 (2.00%) | 3 (1.00%) | 0.53 (0.13-2.05) |  |
|  | Dominant | G/G | 232 (75.10%) | 302 (75.9%) | 1 |  | 301 (77.20%) | 229 (76.30%) | 1 |  |
|  |  | A/G-A/A | 77 (24.90%) | 96 (24.1%) | 0.98 (0.66-1.46) | 0.918 | 89 (22.80%) | 71 (23.70%) | 1.07 (0.74-1.54) | 0.720 |
|  | Recessive | G/G-A/G | 306 (99.00%) | 394 (99.00%) | 1 |  | 382 (98.00%) | 297 (99.00%) | 1 |  |
|  |  | A/A | 3 (1.00%) | 4 (1.00%) | 1.99 (0.30-13.36) | 0.478 | 8 (2.00%) | 3 (1.00%) | 0.51 (0.13-1.99) | 0.320 |
|  | Overdominant | G/G-A/A | 235 (76.00%) | 306 (76.90%) | 1 |  | 309 (79.20%) | 232 (77.30%) | 1 |  |
|  |  | A/G | 74 (23.90%) | 92 (23.10%) | 0.95 (0.63-1.42) | 0.790 | 81 (20.80%) | 68 (22.70%) | 1.13 (0.78-1.64) | 0.510 |
|  | Log-additive | － | － | － | 1.01 (0.69-1.47) | 0.959 | － | － | 1.01 (0.73-1.40) | 0.950 |
| **Gender** |  |  | **Females** | | | | **Males** | | | |
| rs17577 | Codominant | G/G | 184 (75.70%) | 174 (72.20%) | 1 |  | 351 (76.80%) | 355 (77.30%) | 1 |  |
|  |  | A/G | 55 (22.60%) | 65 (27.00%) | 1.20 (0.78-1.84) | 0.510 | 101 (22.10%) | 101 (22.00%) | 1.00 (0.73-1.38) | 0.670 |
|  |  | A/A | 4 (1.60%) | 2 (0.80%) | 0.52 (0.09-3.03) |  | 5 (1.10%) | 3 (0.60%) | 0.52 (0.12-2.25) |  |
|  | Dominant | G/G | 184 (75.70%) | 174 (72.20%) | 1 |  | 351 (76.80%) | 355 (77.30%) | 1 |  |
|  |  | A/G-A/A | 59 (24.30%) | 67 (27.80%) | 1.16 (0.76-1.76) | 0.500 | 106 (23.20%) | 104 (22.70%) | 0.98 (0.71-1.34) | 0.890 |
|  | Recessive | G/G-A/G | 239 (98.30%) | 239 (99.20%) | 1 |  | 452 (98.90%) | 456 (99.30%) | 1 |  |
|  |  | A/A | 4 (1.60%) | 2 (0.80%) | 0.49 (0.08-2.89) | 0.420 | 5 (1.10%) | 3 (0.60%) | 0.52 (0.12-2.24) | 0.370 |
|  | Overdominant | G/G-A/A | 188 (77.40%) | 176 (73.00%) | 1 |  | 356 (77.90%) | 358 (78.00%) | 1 |  |
|  |  | A/G | 55 (22.60%) | 65 (27.00%) | 1.21 (0.79-1.86) | 0.370 | 101 (22.10%) | 101 (22.00%) | 1.01 (0.73-1.39) | 0.950 |
|  | Log-additive | － | － | － | 1.09 (0.74-1.62) | 0.660 | － | － | 0.95 (0.71-1.28) | 0.750 |
| rs13925 | Codominant | G/G | 185 (76.50%) | 175 (72.90%) | 1 |  | 348 (76.20%) | 356 (77.70%) | 1 |  |
|  |  | A/G | 53 (21.90%) | 62 (25.80%) | 1.17 (0.75-1.80) | 0.720 | 102 (22.30%) | 98 (21.40%) | 0.95 (0.69-1.31) | 0.630 |
|  |  | A/A | 4 (1.60%) | 3 (1.20%) | 0.74 (0.16-3.57) |  | 7 (1.50%) | 4 (0.90%) | 0.56 (0.16-1.98) |  |
|  | Dominant | G/G | 185 (76.50%) | 175 (72.90%) | 1 |  | 348 (76.20%) | 356 (77.70%) | 1 |  |
|  |  | A/G-A/A | 57 (23.60%) | 65 (27.10%) | 1.14 (0.74-1.74) | 0.550 | 109 (23.90%) | 102 (22.30%) | 0.92 (0.68-1.27) | 0.630 |
|  | Recessive | G/G-A/G | 238 (98.30%) | 237 (98.80%) | 1 |  | 450 (98.50%) | 454 (99.10%) | 1 |  |
|  |  | A/A | 4 (1.60%) | 3 (1.20%) | 0.72 (0.15-3.42) | 0.670 | 7 (1.50%) | 4 (0.90%) | 0.56 (0.16-2.00) | 0.370 |
|  | Overdominant | G/G-A/A | 189 (78.10%) | 178 (74.20%) | 1 |  | 355 (77.70%) | 360 (78.60%) | 1 |  |
|  |  | A/G | 53 (21.90%) | 62 (25.80%) | 1.17 (0.76-1.81) | 0.470 | 102 (22.30%) | 98 (21.40%) | 0.96 (0.70-1.32) | 0.790 |
|  | Log-additive | － | － | － | 1.09 (0.74-1.61) | 0.660 | － | － | 0.91 (0.68-1.21) | 0.510 |
| **Smoking** |  |  | **Smokers** | | | | **Non- smokers** | | | |
| rs17577 | Codominant | G/G | 255 (75.20%) | 252 (74.80%) | 1 |  | 280 (77.60%) | 277 (76.30%) | 1 |  |
|  |  | A/G | 80 (23.60%) | 83 (24.60%) | 0.97 (0.68-1.40) | 0.740 | 76 (21.10%) | 83 (22.90%) | 1.13 (0.79-1.62) | 0.640 |
|  |  | A/A | 4 (1.20%) | 2 (0.60%) | 0.51 (0.09-2.90) |  | 5 (1.40%) | 3 (0.80%) | 0.62 (0.14-2.70) |  |
|  | Dominant | G/G | 255 (75.20%) | 252 (74.80%) | 1 |  | 280 (77.60%) | 277 (76.30%) | 1 |  |
|  |  | A/G-A/A | 84 (24.80%) | 85 (25.20%) | 0.95 (0.66-1.36) | 0.790 | 81 (22.40%) | 86 (23.70%) | 1.10 (0.77-1.56) | 0.610 |
|  | Recessive | G/G-A/G | 335 (98.80%) | 335 (99.40%) | 1 |  | 356 (98.60%) | 360 (99.20%) | 1 |  |
|  |  | A/A | 4 (1.20%) | 2 (0.60%) | 0.52 (0.09-2.91) | 0.440 | 5 (1.40%) | 3 (0.80%) | 0.61 (0.14-2.62) | 0.500 |
|  | Overdominant | G/G-A/A | 259 (76.40%) | 254 (75.40%) | 1 |  | 285 (79.00%) | 280 (77.10%) | 1 |  |
|  |  | A/G | 80 (23.60%) | 83 (24.60%) | 0.98 (0.68-1.41) | 0.920 | 76 (21.10%) | 83 (22.90%) | 1.14 (0.79-1.63) | 0.490 |
|  | Log-additive | － | － | － | 0.93 (0.66-1.31) | 0.680 | － | － | 1.06 (0.76-1.47) | 0.750 |
| rs13925 | Codominant | G/G | 255 (75.40%) | 253 (75.10%) | 1 |  | 278 (77.00%) | 278 (77.00%) | 1 |  |
|  |  | A/G | 78 (23.10%) | 80 (23.70%) | 0.97 (0.67-1.41) | 0.910 | 77 (21.30%) | 80 (22.20%) | 1.06 (0.74-1.52) | 0.580 |
|  |  | A/A | 5 (1.50%) | 4 (1.20%) | 0.75 (0.19-2.93) |  | 6 (1.70%) | 3 (0.80%) | 0.50 (0.12-2.06) |  |
|  | Dominant | G/G | 255 (75.40%) | 253 (75.10%) | 1 |  | 278 (77.00%) | 278 (77.00%) | 1 |  |
|  |  | A/G-A/A | 83 (24.60%) | 84 (24.90%) | 0.96 (0.67-1.38) | 0.820 | 83 (23.00%) | 83 (23.00%) | 1.02 (0.71-1.45) | 0.930 |
|  | Recessive | G/G-A/G | 333 (98.50%) | 333 (98.80%) | 1 |  | 355 (98.30%) | 358 (99.20%) | 1 |  |
|  |  | A/A | 5 (1.50%) | 4 (1.20%) | 0.76 (0.20-2.94) | 0.690 | 6 (1.70%) | 3 (0.80%) | 0.49 (0.12-2.03) | 0.310 |
|  | Overdominant | G/G-A/A | 260 (76.90%) | 257 (76.30%) | 1 |  | 284 (78.70%) | 281 (77.80%) | 1 |  |
|  |  | A/G | 78 (23.10%) | 80 (23.70%) | 0.98 (0.68-1.41) | 0.910 | 77 (21.30%) | 80 (22.20%) | 1.07 (0.74-1.53) | 0.720 |
|  | Log-additive | － | － | － | 0.95 (0.68-1.32) | 0.760 | － | － | 0.97 (0.70-1.35) | 0.870 |
| **Drinking** |  |  | **Drinkers** | | | | **Non-drinkers** | | | |
| rs17577 | Codominant | G/G | 272 (78.80%) | 250 (74.20%) | 1 |  | 263 (74.10%) | 279 (76.90%) | 1 |  |
|  |  | A/G | 69 (20.00%) | 84 (24.90%) | 1.31 (0.90-1.91) | 0.330 | 87 (24.50%) | 82 (22.60%) | 0.86 (0.60-1.22) | 0.390 |
|  |  | A/A | 4 (1.20%) | 3 (0.90%) | 0.78 (0.17-3.58) |  | 5 (1.40%) | 2 (0.60%) | 0.40 (0.07-2.17) |  |
|  | Dominant | G/G | 272 (78.80%) | 250 (74.20%) | 1 |  | 263 (74.10%) | 279 (76.90%) | 1 |  |
|  |  | A/G-A/A | 73 (21.20%) | 87 (25.80%) | 1.28 (0.89-1.85) | 0.180 | 92 (25.90%) | 84 (23.10%) | 0.83 (0.59-1.18) | 0.300 |
|  | Recessive | G/G-A/G | 341 (98.80%) | 334 (99.10%) | 1 |  | 350 (98.60%) | 361 (99.50%) | 1 |  |
|  |  | A/A | 4 (1.20%) | 3 (0.90%) | 0.73 (0.16-3.36) | 0.690 | 5 (1.40%) | 2 (0.60%) | 0.41 (0.08-2.24) | 0.280 |
|  | Overdominant | G/G-A/A | 276 (80.00%) | 253 (75.10%) | 1 |  | 268 (75.50%) | 281 (77.40%) | 1 |  |
|  |  | A/G | 69 (20.00%) | 84 (24.90%) | 1.32 (0.91-1.91) | 0.150 | 87 (24.50%) | 82 (22.60%) | 0.86 (0.61-1.23) | 0.420 |
|  | Log-additive | － | － | － | 1.22 (0.87-1.72) | 0.250 | － | － | 0.82 (0.59-1.14) | 0.230 |
| rs13925 | Codominant | G/G | 270 (78.30%) | 250 (74.40%) | 1 |  | 263 (74.30%) | 281 (77.60%) | 1 |  |
|  |  | A/G | 69 (20.00%) | 83 (24.70%) | 1.29 (0.89-1.87) | 0.260 | 86 (24.30%) | 77 (21.30%) | 0.81 (0.57-1.16) | 0.480 |
|  |  | A/A | 6 (1.70%) | 3 (0.90%) | 0.54 (0.13-2.24) |  | 5 (1.40%) | 4 (1.10%) | 0.73 (0.19-2.86) |  |
|  | Dominant | G/G | 270 (78.30%) | 250 (74.40%) | 1 |  | 263 (74.30%) | 281 (77.60%) | 1 |  |
|  |  | A/G-A/A | 75 (21.70%) | 86 (25.60%) | 1.23 (0.85-1.77) | 0.270 | 91 (25.70%) | 81 (22.40%) | 0.81 (0.57-1.15) | 0.230 |
|  | Recessive | G/G-A/G | 339 (98.30%) | 333 (99.10%) | 1 |  | 349 (98.60%) | 358 (98.90%) | 1 |  |
|  |  | A/A | 6 (1.70%) | 3 (0.90%) | 0.51 (0.12-2.10) | 0.340 | 5 (1.40%) | 4 (1.10%) | 0.76 (0.20-2.99) | 0.700 |
|  | Overdominant | G/G-A/A | 276 (80.00%) | 253 (75.30%) | 1 |  | 268 (75.70%) | 285 (78.70%) | 1 |  |
|  |  | A/G | 69 (20.00%) | 83 (24.70%) | 1.30 (0.90-1.89) | 0.170 | 86 (24.30%) | 77 (21.30%) | 0.81 (0.57-1.17) | 0.260 |
|  | Log-additive | － | － | － | 1.14 (0.82-1.59) | 0.440 | － | － | 0.82 (0.59-1.14) | 0.230 |

Abbreviations: IS, ischemic stroke; SNPs, single nucleotide polymorphisms; OR, odds ratio; CI, confidence interval.

Note: *p* < 0.05 indicates statistical significance.

**Supplementary table 4** Correlations between *MMP-9* gene polymorphisms and IS susceptibility stratified by complications (hypertension and diabetes)

| **SNP ID** | **Model** | **Genotype** | **Control** | **Case1** | **Case2** | **OR (95% CI)** | **P** | **OR (95% CI)** | **P** | **OR (95% CI)** | **P** |
| --- | --- | --- | --- | --- | --- | --- | --- | --- | --- | --- | --- |
|  |  |  |  | **IS with HYP** | **IS without HYP** | **IS with HYP vs Control** | | **IS without HYP vs Control** | | **IS with HYP vs Stroke without HYP** | |
|  | Codominant | G/G | 535 (76.4%) | 357 (77.4%) | 172 (72%) | 1 | 0.760 | 1 | 0.110 | 1 | 0.200 |
| rs17577 |  | A/G | 156 (22.3%) | 100 (21.7%) | 66 (27.6%) | 0.97 (0.73-1.29) |  | 1.32 (0.95-1.85) |  | 0.73 (0.51-1.05) |  |
|  |  | A/A | 9 (1.3%) | 4 (0.9%) | 1 (0.4%) | 0.66 (0.20-2.15) |  | 0.32 (0.04-2.58) |  | 1.86 (0.21-16.84) |  |
|  | Dominant | G/G | 535 (76.4%) | 357 (77.4%) | 172 (72%) | 1 | 0.730 | 1 | 0.170 | 1 | 0.120 |
|  |  | A/G-A/A | 165 (23.6%) | 104 (22.6%) | 67 (28%) | 0.95 (0.72-1.26) |  | 1.27 (0.91-1.77) |  | 0.75 (0.52-1.08) |  |
|  | Recessive | G/G-A/G | 691 (98.7%) | 457 (99.1%) | 238 (99.6%) | 1 | 0.480 | 1 | 0.190 | 1 | 0.510 |
|  |  | A/A | 9 (1.3%) | 4 (0.9%) | 1 (0.4%) | 0.66 (0.20-2.16) |  | 0.30 (0.04-2.40) |  | 2.01 (0.22-18.18) |  |
|  | Overdominant | G/G-A/A | 544 (77.7%) | 361 (78.3%) | 173 (72.4%) | 1 | 0.860 | 1 | 0.092 | 1 | 0.090 |
|  |  | A/G | 156 (22.3%) | 100 (21.7%) | 66 (27.6%) | 0.97 (0.73-1.30) |  | 1.34 (0.96-1.88) |  | 0.73 (0.51-1.05) |  |
|  | Log-additive | --- | --- | --- | --- | 0.94 (0.72-1.22) | 0.630 | 1.18 (0.86-1.61) | 0.310 | 0.79 (0.56-1.11) | 0.170 |
|  | Codominant | G/G | 533 (76.2%) | 358 (77.8%) | 173 (72.7%) | 1 | 0.820 | 1 | 0.110 | 1 | 0.110 |
| rs13925 |  | A/G | 155 (22.2%) | 96 (20.9%) | 64 (26.9%) | 0.93 (0.70-1.24) |  | 1.28 (0.91-1.80) |  | 0.72 (0.50-1.05) |  |
|  |  | A/A | 11 (1.6%) | 6 (1.3%) | 1 (0.4%) | 0.82 (0.30-2.23) |  | 0.28 (0.04-2.19) |  | 2.95 (0.35-24.79) |  |
|  | Dominant | G/G | 533 (76.2%) | 358 (77.8%) | 173 (72.7%) | 1 | 0.570 | 1 | 0.260 | 1 | 0.140 |
|  |  | A/G-A/A | 166 (23.8%) | 102 (22.2%) | 65 (27.3%) | 0.92 (0.70-1.22) |  | 1.22 (0.87-1.70) |  | 0.76 (0.53-1.09) |  |
|  | Recessive | G/G-A/G | 688 (98.4%) | 454 (98.7%) | 237 (99.6%) | 1 | 0.710 | 1 | 0.130 | 1 | 0.220 |
|  |  | A/A | 11 (1.6%) | 6 (1.3%) | 1 (0.4%) | 0.83 (0.30-2.26) |  | 0.26 (0.03-2.06) |  | 3.19 (0.38-26.77) |  |
|  | Overdominant | G/G-A/A | 544 (77.8%) | 364 (79.1%) | 174 (73.1%) | 1 | 0.630 | 1 | 0.130 | 1 | 0.076 |
|  |  | A/G | 155 (22.2%) | 96 (20.9%) | 64 (26.9%) | 0.93 (0.70-1.24) |  | 1.30 (0.93-1.83) |  | 0.72 (0.50-1.03) |  |
|  | Log-additive | --- | --- | --- | --- | 0.92 (0.71-1.19) | 0.540 | 1.12 (0.82-1.53) | 0.470 | 0.82 (0.58-1.15) | 0.250 |
|  |  |  |  | **IS with diabetes** | **IS without diabetes** | **IS with diabetes vs Control** | | **IS without diabetes vs Control** | | **IS with diabetes vs IS without diabetes** | |
|  | Codominant | G/G | 535 (76.4%) | 132 (78.1%) | 397 (74.8%) | 1 | 0.750 | 1 | 0.100 | 1 | 0.120 |
| rs17577 |  | A/G | 156 (22.3%) | 34 (20.1%) | 132 (24.9%) | 0.89 (0.58-1.35) |  | 1.15 (0.88-1.51) |  | 0.76 (0.50-1.18) |  |
|  |  | A/A | 9 (1.3%) | 3 (1.8%) | 2 (0.4%) | 1.39 (0.37-5.22) |  | 0.28 (0.06-1.32) |  | 4.20 (0.68-25.74) |  |
|  | Dominant | G/G | 535 (76.4%) | 132 (78.1%) | 397 (74.8%) | 1 | 0.660 | 1 | 0.470 | 1 | 0.350 |
|  |  | A/G-A/A | 165 (23.6%) | 37 (21.9%) | 134 (25.2%) | 0.91 (0.61-1.37) |  | 1.10 (0.85-1.44) |  | 0.82 (0.54-1.25) |  |
|  | Recessive | G/G-A/G | 691 (98.7%) | 166 (98.2%) | 529 (99.6%) | 1 | 0.610 | 1 | 0.064 | 1 | 0.100 |
|  |  | A/A | 9 (1.3%) | 3 (1.8%) | 2 (0.4%) | 1.42 (0.38-5.34) |  | 0.27 (0.06-1.27) |  | 4.47 (0.73-27.34) |  |
|  | Overdominant | G/G-A/A | 544 (77.7%) | 135 (79.9%) | 399 (75.1%) | 1 | 0.550 | 1 | 0.260 | 1 | 0.190 |
|  |  | A/G | 156 (22.3%) | 34 (20.1%) | 132 (24.9%) | 0.88 (0.58-1.34) |  | 1.17 (0.89-1.52) |  | 0.75 (0.49-1.16) |  |
|  | Log-additive | --- | --- | --- | --- | 0.95 (0.66-1.38) | 0.790 | 1.04 (0.81-1.34) | 0.750 | 0.89 (0.60-1.33) | 0.580 |
|  | Codominant | G/G | 533 (76.2%) | 132 (78.1%) | 399 (75.4%) | 1 | 0.620 | 1 | 0.180 | 1 | 0.084 |
| rs13925 |  | A/G | 155 (22.2%) | 33 (19.5%) | 127 (24%) | 0.86 (0.56-1.31) |  | 1.11 (0.84-1.45) |  | 0.77 (0.50-1.19) |  |
|  |  | A/A | 11 (1.6%) | 4 (2.4%) | 3 (0.6%) | 1.48 (0.46-4.74) |  | 0.37 (0.10-1.33) |  | 4.12 (0.88-19.26) |  |
|  | Dominant | G/G | 533 (76.2%) | 132 (78.1%) | 399 (75.4%) | 1 | 0.620 | 1 | 0.680 | 1 | 0.420 |
|  |  | A/G-A/A | 166 (23.8%) | 37 (21.9%) | 130 (24.6%) | 0.90 (0.60-1.35) |  | 1.06 (0.81-1.38) |  | 0.84 (0.55-1.28) |  |
|  | Recessive | G/G-A/G | 688 (98.4%) | 165 (97.6%) | 526 (99.4%) | 1 | 0.490 | 1 | 0.091 | 1 | 0.061 |
|  |  | A/A | 11 (1.6%) | 4 (2.4%) | 3 (0.6%) | 1.53 (0.48-4.88) |  | 0.36 (0.10-1.30) |  | 4.37 (0.94-20.37) |  |
|  | Overdominant | G/G-A/A | 544 (77.8%) | 136 (80.5%) | 402 (76%) | 1 | 0.460 | 1 | 0.410 | 1 | 0.190 |
|  |  | A/G | 155 (22.2%) | 33 (19.5%) | 127 (24%) | 0.85 (0.56-1.30) |  | 1.12 (0.86-1.47) |  | 0.75 (0.48-1.16) |  |
|  | Log-additive | --- | --- | --- | --- | 0.96 (0.67-1.37) | 0.800 | 1.00 (0.78-1.28) | 0.990 | 0.94 (0.64-1.39) | 0.760 |

Abbreviations: IS, ischemic stroke; SNPs, single nucleotide polymorphisms; OR, odds ratio; CI, confidence interval.

Note: *p* < 0.05 indicates statistical significance.
